# MEK inhibitors increase the mortality rate in mice with LPS-induced inflammation through IL-12-NO signaling

**DOI:** 10.1038/s41420-023-01674-w

**Published:** 2023-10-13

**Authors:** Ryota Hashimoto, Hiroshi Koide, Youichi Katoh

**Affiliations:** 1 Laboratory of Cell Biology, Biomedical Research Core Facilities, Juntendo University Graduate School of Medicine, Hongo 2-1-1, Bunkyo-ku, Tokyo, 113-8421 Japan; 2 Department of Physiology, Juntendo University Faculty of Medicine, Hongo 2-1-1, Bunkyo-ku, Tokyo, 113-8421 Japan; 3 Laboratory of Molecular and Biochemical Research, Biomedical Research Core Facilities, Juntendo University Graduate School of Medicine, Hongo 2-1-1, Bunkyo-ku, Tokyo, 113-8421 Japan; 4 Department of Cardiovascular Biology and Medicine, Juntendo University Graduate School of Medicine, Hongo 2-1-1, Bunkyo-ku, Tokyo, 113-8421 Japan; 5 Juntendo University Faculty of International Liberal Arts, Hongo 2-1-1, Bunkyo-ku, Tokyo, 112-8421 Japan

**Keywords:** Acute inflammation, Drug safety

## Abstract

Lipopolysaccharide (LPS) is an endotoxin that can cause an acute inflammatory response. Nitric oxide (NO) is one of the most important innate immune system components and is synthesized by inducible NOS (iNOS) in macrophages in response to stimulation with LPS. LPS activates the RAS-RAF-mitogen-activated protein kinase/ERK kinase (MEK)-extracellular-signal-regulated kinase (ERK) signaling cascade in macrophages. The purpose of this study was to examine how the combination of LPS and MEK inhibitors, which have been used as anticancer agents in recent years, affects inflammation. We showed that MEK inhibitors enhanced iNOS expression and NO production in LPS-stimulated mouse bone marrow-derived macrophages. A MEK inhibitor increased the mortality rate in mice with LPS-induced inflammation. The expression of the cytokine interleukin-12 (IL-12) in macrophages was enhanced by the MEK inhibitor, as shown by a cytokine array and ELISA. IL-12 enhanced iNOS expression and NO production in response to LPS. We also showed that tumor necrosis factor (TNF-α) was secreted by macrophage after stimulation with LPS and that TNF-α and IL-12 synergistically induced iNOS expression and NO production. An anti-IL-12 neutralizing antibody prevented NO production and mortality in an LPS-induced inflammation mouse model in the presence of a MEK inhibitor. These results suggest that the MEK inhibitor increases the mortality rate in mice with LPS-induced inflammation through IL-12-NO signaling.

## Introduction

Cancer immunotherapies, including checkpoint inhibitors and adoptive cell therapy, use certain parts of a patient’s immune system to attack cancer cells [1, 2]. Bacteria-mediated cancer therapy, which is a cancer immunotherapy, has been studied for a century, and attenuated Salmonella is a promising therapeutic agent [3, 4]. Salmonella is a gram-negative bacterium, and lipopolysaccharide (LPS) is an important outer membrane component of gram-negative bacteria. Mouse experiments have shown that LPS has cancer-suppressing effects [5, 6] and promotes metastasis [7, 8]. The combination of the anticancer agent doxorubicin and LPS has been reported to be more effective in prolonging life in mice implanted with cancer than the administration of doxorubicin alone [9]. The effects of the combination of LPS and other anticancer agents remain unknown.

Mitogen-activated protein kinases (MAPKs) include the extracellular signal-regulated kinase (ERK1/2), Jun amino-terminal kinase (JNK1/2/3), and p38 MAPK (p38) subfamilies, which are crucial regulators of cellular physiology and pathology. The RAS–RAF–MAPK/ERK kinase (MEK)–ERK signaling cascade is among the most frequently mutated pathways in human cancers [10]. Mutations in the BRAF gene, which is a member of the RAF family, have been identified in patients with skin cancer (malignant melanoma), thyroid cancer, colon cancer, and lung cancer [11]; thus, BRAF inhibitors and MEK inhibitors, which target the pathway downstream of BRAF, have been used as anticancer agents in recent years [12, 13]. We and others have reported that ERK is activated by LPS [14–16]. LPS can also cause an acute inflammatory response [17–19]. Nitric oxide (NO) is one of the most important innate immune system components and is synthesized by inducible NOS (iNOS, NOS2) in macrophages for several hours after stimulation with LPS [20, 21].

The purpose of this study was to examine how the combination of MEK inhibitors and LPS affects inflammation. In the present study, we showed that MEK inhibitors enhanced iNOS expression and NO production in response to LPS through interleukin-12 (IL-12) induction in mouse bone marrow-derived macrophages. We identified IL-12 as a pivotal cytokine that enhances iNOS expression and NO production in combination with tumor necrosis factor (TNF-α). We also showed that MEK inhibitors increased the mortality rate in mice with LPS-induced inflammation through IL-12-NO signaling.

## Results

### LPS-induced NO production in macrophages is enhanced by MEK inhibitors

**Fig. 1:**
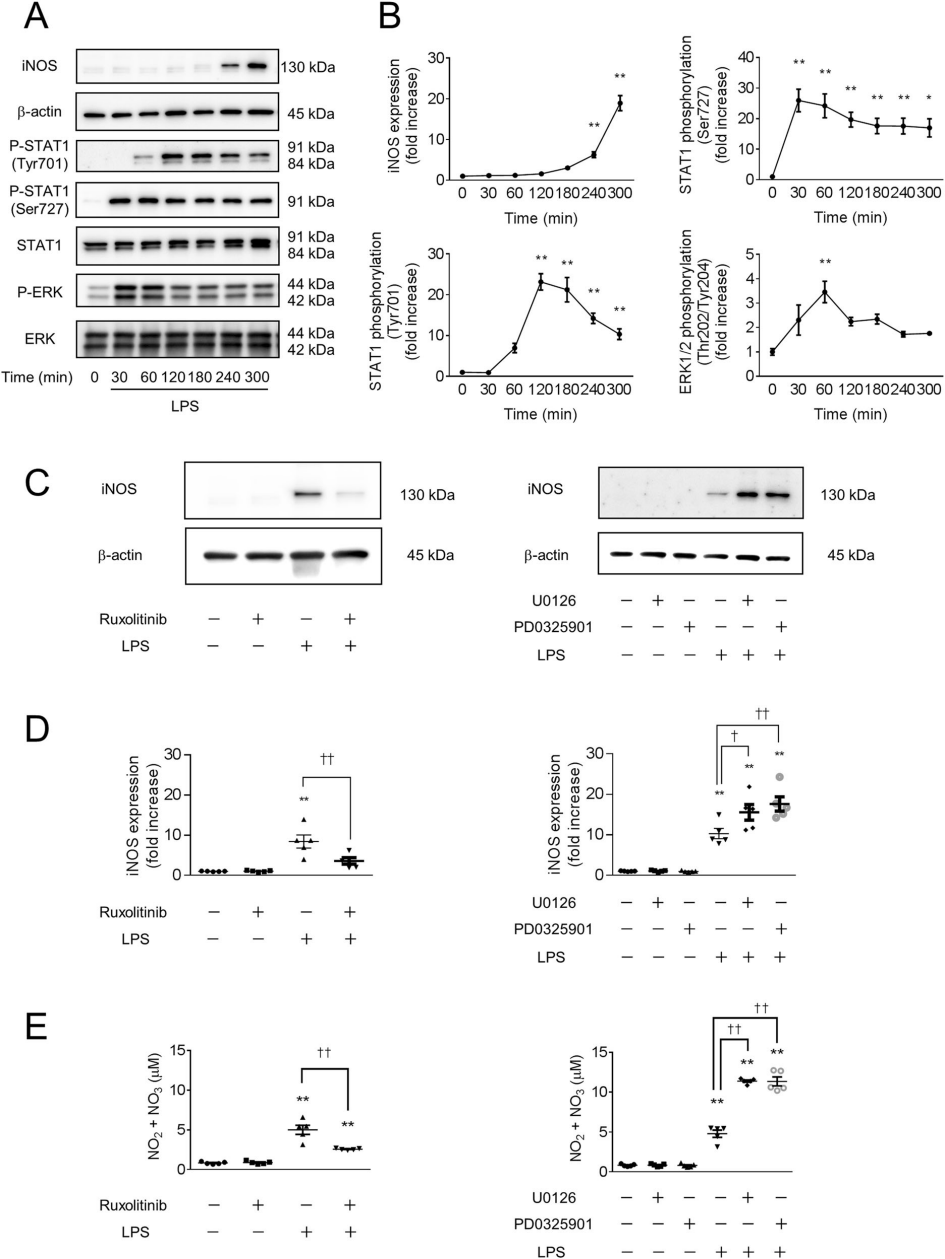
LPS-induced iNOS expression in mouse macrophages is suppressed by a JAK-STAT inhibitor and enhanced by a MEK inhibitor. **A**, **B** Bone marrow-derived macrophages (BMDMs) were treated with 100 ng/mL lipopolysaccharide (LPS). The cell extracts were sampled at different time points as indicated after LPS stimulation and analyzed by western blotting. After LPS stimulation, increased phosphorylation of ERK1/2 (Thr202/Tyr204), STAT1 (Ser727), STAT1 (Tyr701), and inducible nitric oxide synthase (iNOS) were detected. *n* = 5 (*p < 0.05, **p < 0.01 vs. pretreatment (0 min) group). **C**–**E** BMDMs were treated with inhibitors of JAK-STAT (1 μM ruxolitinib) and MAPK/MEK (1 μM U0126 or 10 nM PD0325901) for 60 min, followed by treatment with 100 ng/mL LPS for 300 min (**C**, **D**) or overnight (**E**). **C**, **D** The cell extracts were analyzed by western blotting. LPS-induced iNOS expression was suppressed by a JAK-STAT inhibitor and enhanced by a MEK inhibitor. **E** The concentration of NO in the supernatant was determined using 2,3-diaminonaphthalene (DAN) and is shown as the concentration of NO_2_^−^ + NO_3_^−^. **C**–**E** *n* = 5 (**p < 0.01 vs. the control group; ^†^*p* < 0.05, ^††^*p* < 0.01 vs. the LPS-treated group).

We first examined the effects of LPS on the Ras/Raf/MEK/ERK pathway and JAK-STAT pathway in macrophages. The phosphorylation of signal transduction and activator of transcription 1 (STAT1) (Ser727) was induced after 30 min, and ERK1/2 (Thr202/Tyr204) was induced after 60 min of treatment with 100 ng/mL LPS. In contrast, the phosphorylation of STAT1 (Tyr701), which is critical for iNOS expression in macrophages [22–24], was induced after 120 min of treatment and continued for at least 180 min (Fig. 1A, B). These results suggest that ERK1/2 (Thr202/Tyr204) is phosphorylated before STAT1 (Tyr701) in LPS-induced mouse bone marrow-derived macrophages.

The expression of iNOS was detected after 240 min of LPS stimulation (Fig. 1A, B). Pretreatment with the JAK-STAT inhibitor ruxolitinib (1 μM) blocked LPS-induced iNOS expression (Fig. 1C, D) and NO production (Fig. 1E). However, pretreatment with the MEK inhibitors U0126 (1 μM) and PD0325901 (10 nM) increased iNOS expression in LPS-stimulated macrophages (Fig 1C, D). Similarly, pretreatment with MEK inhibitors increased NO production in LPS-stimulated macrophages (Fig. 1E). These results suggest that MEK inhibition enhances iNOS expression and NO production in LPS-stimulated mouse bone marrow-derived macrophages.

### Enhanced iNOS expression and NO production in response to stimulation with LPS and a MEK inhibitor are suppressed by inhibition of the JAK-STAT pathway in macrophages

**Fig. 2:**
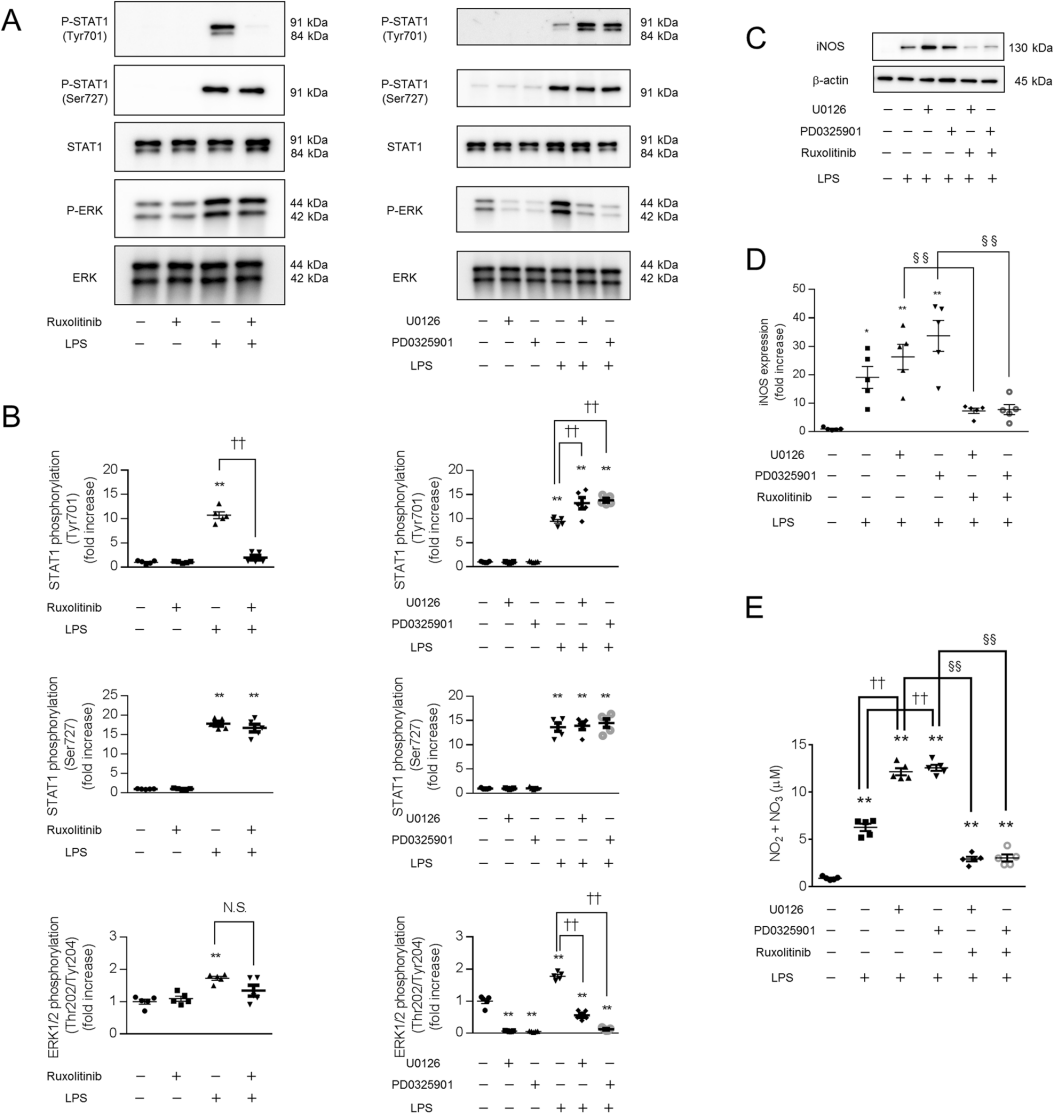
Enhanced iNOS expression and NO production in LPS-stimulated macrophages treated with a MEK inhibitor were blocked by a JAK-STAT inhibitor. BMDMs were treated with JAK-STAT and MEK inhibitors for 60 min, followed by stimulation with LPS for 60 min, 300 min (**A**–**D**) or overnight (**E**). **A**–**D** The cell extracts were analyzed by western blotting. **E** The concentration of NO in the supernatant is shown as the concentration of NO_2_^−^ + NO_3_^−^. **A**, **B** Pretreatment with the MEK inhibitor enhanced the phosphorylation of STAT1 (Tyr701) after stimulation with LPS. **C**–**E** Enhanced iNOS expression and NO production in response to stimulation with LPS and a MEK inhibitor were blocked by a JAK-STAT inhibitor. **A**–**E** *n* = 5 (*p < 0.05, **p < 0.01 vs. the control group; ^††^*p* < 0.01 vs. the LPS-treated group; ^§§^*p* < 0.01).

We investigated the relationship between the Ras/Raf/MEK/ERK pathway and the JAK-STAT pathway in macrophages. Pretreatment with the MEK inhibitors U0126 (1 μM) and PD0325901 (10 nM) blocked ERK phosphorylation after 60 min of LPS stimulation; however, these inhibitors increased STAT1 phosphorylation at Tyr701 but not Ser727 after 300 min of LPS stimulation (Fig. 2A, B). Pretreatment with the JAK-STAT inhibitor ruxolitinib (1 μM) did not affect ERK phosphorylation after 60 min of LPS stimulation, and this inhibitor blocked the phosphorylation of STAT1 at Tyr701 but not Ser727 after 300 min of LPS stimulation (Fig 2A, B).

We next investigated the effects of JAK-STAT inhibitors on the enhanced iNOS expression and NO production during stimulation with LPS and MEK inhibitors. Pretreatment with the JAK-STAT inhibitor ruxolitinib (1 μM) blocked the increases in iNOS expression (Fig. 2C, D) and NO production (Fig. 2E) during stimulation with LPS and the MEK inhibitor U0126 (1 μM). Similarly, ruxolitinib (1 μM) blocked the increases in iNOS expression (Fig. 2C, D) and NO production (Fig. 2E) during stimulation with LPS and the MEK inhibitor PD0325901 (10 nM). These results suggest that the MEK inhibitor enhances iNOS expression and NO production in LPS-stimulated mouse bone marrow-derived macrophages by enhancing activation of the JAK-STAT pathway.

### More IL-12 is secreted during stimulation with LPS and a MEK inhibitor in macrophages

**Fig. 3:**
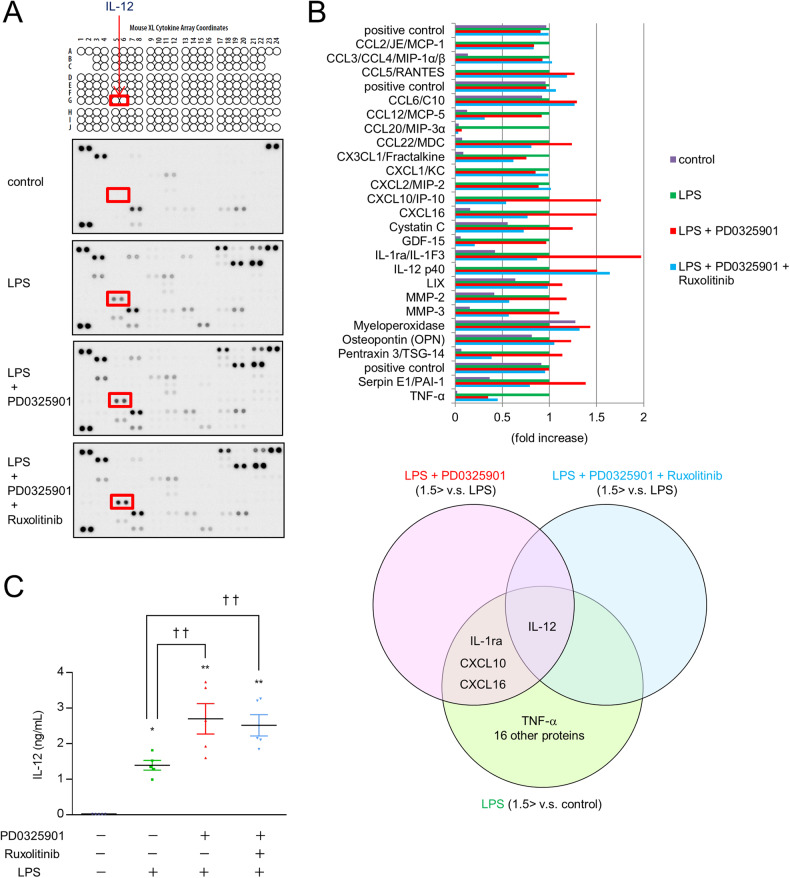
Increased IL-12 secretion in response to stimulation with LPS and a MEK inhibitor. **A**–**C** BMDMs were treated with the MEK inhibitor PD0325901 and the JAK-STAT inhibitor ruxolitinib for 60 min, followed by stimulation with LPS for 300 min. A membrane-based multiplex antibody array was used to determine the relative levels of secreted cytokines in the supernatant. **A** Representative blots of the antibody array are shown. **B** The bar graph shows the densitometric analysis (*n* = 1). IL-12 was over 1.5 times more abundant in the LPS + PD0325901 group and the LPS + PD0325901 + ruxolitinib group than in the LPS-treated group. **C** The concentration of IL-12 in the supernatant was determined by ELISA. *n* = 5 (*p < 0.05, **p < 0.01 vs. the control group; ^††^*p* < 0.01 vs. the LPS-treated group).

We investigated the cytokine that mediates the interaction between the Ras/Raf/MEK/ERK pathway and the JAK-STAT pathway in macrophages using an antibody array and ELISA. Antibody array membrane analysis showed basal expression of several cytokines in macrophages. LPS caused an increase in the expression of several cytokines (21 cytokines, including IL-12 and TNF-α, were at least 1.5 times more abundant in the treatment group than in the control group, Fig. 3A, B). We hypothesized that the secretion of specific cytokines is increased by LPS, increased by stimulation with LPS and MEK inhibitors, and not changed by JAK-STAT inhibitors. We chose IL-12 as a candidate mediator of the interaction between the Ras/Raf/MEK/ERK pathway and the JAK-STAT pathway. IL-12, IL-1ra, CXCL10, and CXCL16 levels in the LPS plus MEK inhibitor group were over 1.5 times higher than those in the LPS group. However, only the IL-12 levels were unaffected by additional treatment with a JAK-STAT inhibitor (Fig. 3A, B). ELISA showed that the secretion of IL-12 was increased by stimulation with LPS and a MEK inhibitor, and this secretion was not changed by additional treatment with a JAK-STAT inhibitor (Fig. 3C). These results suggest that there is an increase in IL-12 secretion by macrophages in response to stimulation with LPS and a MEK inhibitor.

### IL-12 enhances iNOS expression and NO production in LPS-stimulated macrophages

**Fig. 4:**
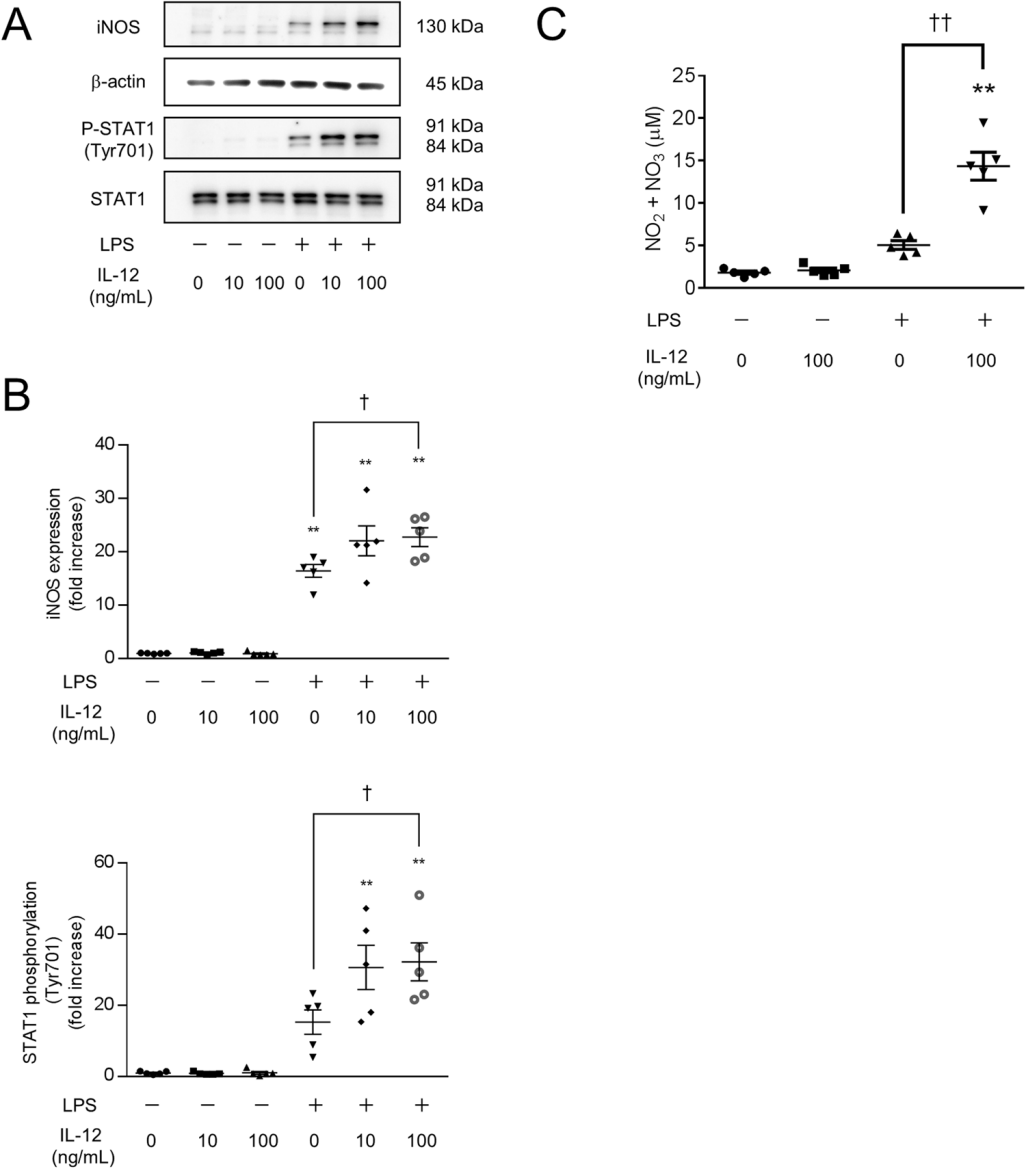
IL-12 enhances iNOS expression and NO production in LPS-stimulated macrophages. BMDMs were treated with the indicated concentrations of IL-12 and LPS for 300 min (**A**, **B**) or overnight (**C**). **A**, **B** The cell extracts were analyzed by western blotting. **C** The concentration of NO in the supernatant is shown as the concentration of NO_2_^−^ + NO_3_^−^. **A**–**C** IL-12 enhanced the phosphorylation of STAT1 (Tyr701), iNOS expression, and NO production in response to stimulation with LPS. *n* = 5 (**p < 0.01 vs. the control group; ^†^*p* < 0.05, ^††^*p* < 0.01 vs. the LPS-treated group).

We examined the effects of IL-12 on macrophages. The phosphorylation of STAT1 (Tyr701) was not changed after 300 min of IL-12 treatment; however, the phosphorylation of STAT1 (Tyr701) was increased after 300 min of stimulation with LPS and IL-12 (100 ng/mL, Fig. 4A, B). The expression of iNOS was not changed after 300 min of IL-12 treatment; however, the expression of iNOS was increased after 300 min of stimulation with LPS and IL-12 (100 ng/mL, Fig. 4A, B). Similarly, overnight IL-12 treatment did not affect NO production, while overnight stimulation with LPS and IL-12 (100 ng/mL) enhanced NO production (Fig. 4C). These results suggest that IL-12 enhances iNOS expression and NO production in LPS-induced mouse bone marrow-derived macrophages.

### IL-12 enhances iNOS expression and NO production in the presence of TNF-α in macrophages

**Fig. 5:**
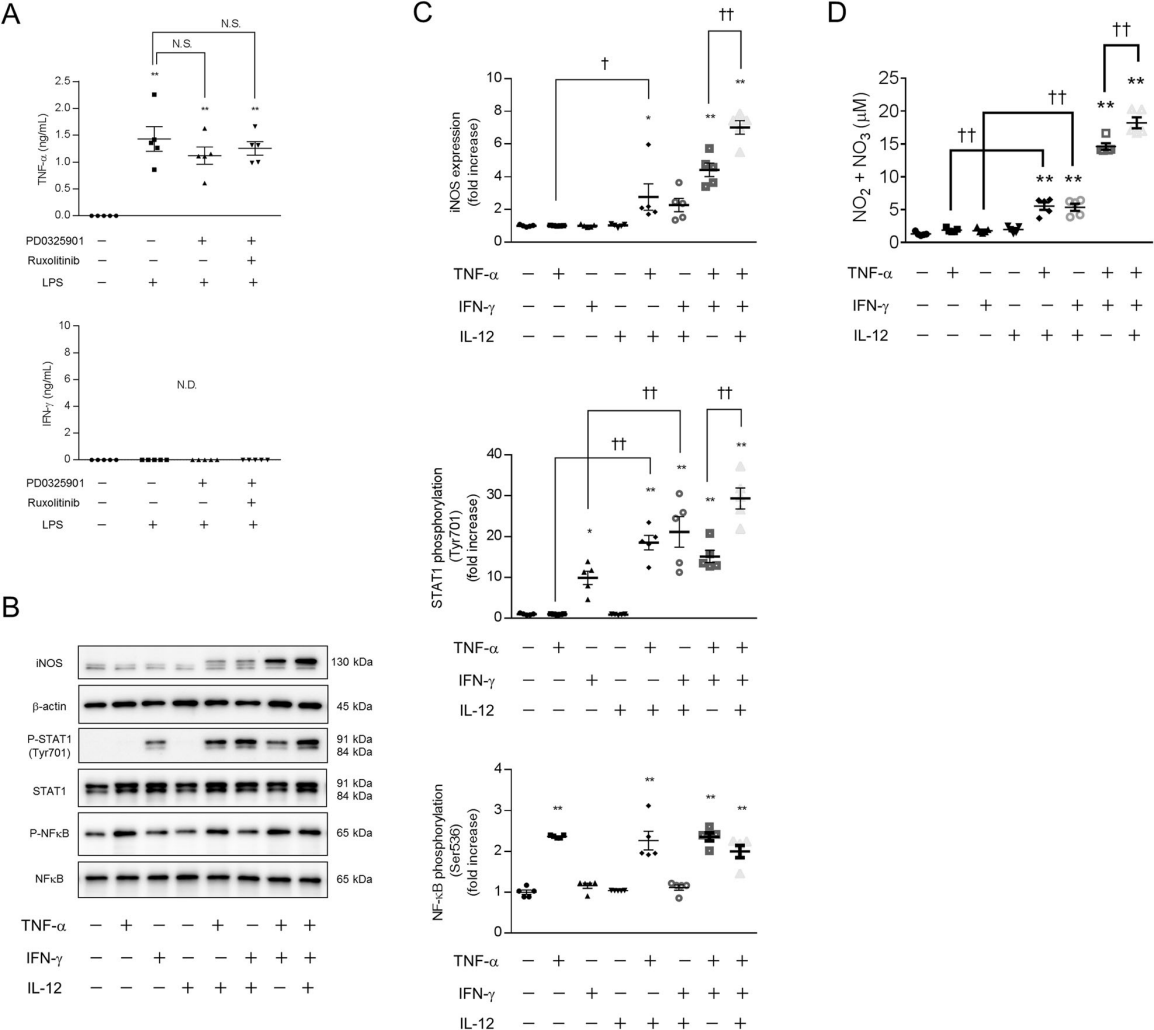
IL-12 enhances iNOS expression and NO production in TNF-α−treated macrophages. **A** BMDMs were treated with a MEK inhibitor and a JAK-STAT inhibitor for 60 min, followed by stimulation with LPS for 300 min. The concentrations of TNF-α and IFN-γ in the supernatant were determined by ELISA. TNF-α secretion was increased by LPS. **B**–**D** BMDMs were treated with 100 ng/mL IL-12 in the presence of TNF-α (30 ng/mL), IFN-γ (30 ng/mL), or TNF-α plus IFN-γ for 300 min (**B**, **C**) or overnight (**D**). **B**, **C** The cell extracts were analyzed by western blotting. **D** The concentration of NO in the supernatant is shown as the concentration of NO_2_^−^ + NO_3_^−^. **B**–**D** IL-12 enhanced the phosphorylation of STAT1 (Tyr701), iNOS expression, and NO production in the presence of TNF-α. **A**–**D** *n* = 5 (*p < 0.05, **p < 0.01 vs. the control group; ^†^*p* < 0.05, ^††^*p* < 0.01). N.S. means not significant, and N.D. means not detected.

We identified the cytokine that coordinates with IL-12 to enhance STAT1 phosphorylation (Tyr701) and iNOS expression. TNF-α and interferon (IFN)-γ are key mediators of iNOS expression and NO production [25, 26]; thus, we investigated the secretion of these cytokines by macrophages using ELISA. We did not detect IFN-γ secretion (Fig. 5A). On the other hand, the secretion of TNF-α was increased by stimulation with LPS for 300 min (Fig. 5A). Notably, the MEK inhibitor PD0325901 (10 nM) did not affect the secretion of TNF-α (Fig. 5A).

We next examined the effects of IL-12 on macrophages in the presence of TNF-α, IFN-γ, or TNF-α plus IFN-γ. The phosphorylation of STAT1 (Tyr701) was not altered after 300 min of IL-12 treatment (100 ng/mL); however, IL-12 enhanced the phosphorylation of STAT1 (Tyr701) in the presence of TNF-α (30 ng/mL), IFN-γ (30 ng/mL), or TNF-α plus IFN-γ (Fig. 5B, C). The nuclear factor κB (NF-κB) pathway is an important pathway associated with inflammation and iNOS expression in macrophages [27, 28]. Although TNF-α increased the phosphorylation of NF-κB (Ser536), IL-12 did not affect the phosphorylation of NF-κB (Fig. 5B, C). The expression of iNOS was not changed after 300 min of IL-12 treatment; however, IL-12 enhanced the expression of iNOS in the presence of TNF-α or TNF-α plus IFN-γ (Fig. 5B, C). Similarly, overnight treatment with IL-12 did not affect NO production, while IL-12 enhanced NO production in the presence of TNF-α, IFN-γ, or TNF-α plus IFN-γ (Fig. 5D). These results suggest that IL-12 enhances iNOS expression and NO production in mouse bone marrow-derived macrophages in the presence of TNF-α

### The increases in iNOS expression and NO production in LPS-stimulated macrophages treated with a MEK inhibitor are blocked by an anti-IL-12 neutralizing antibody

**Fig. 6:**
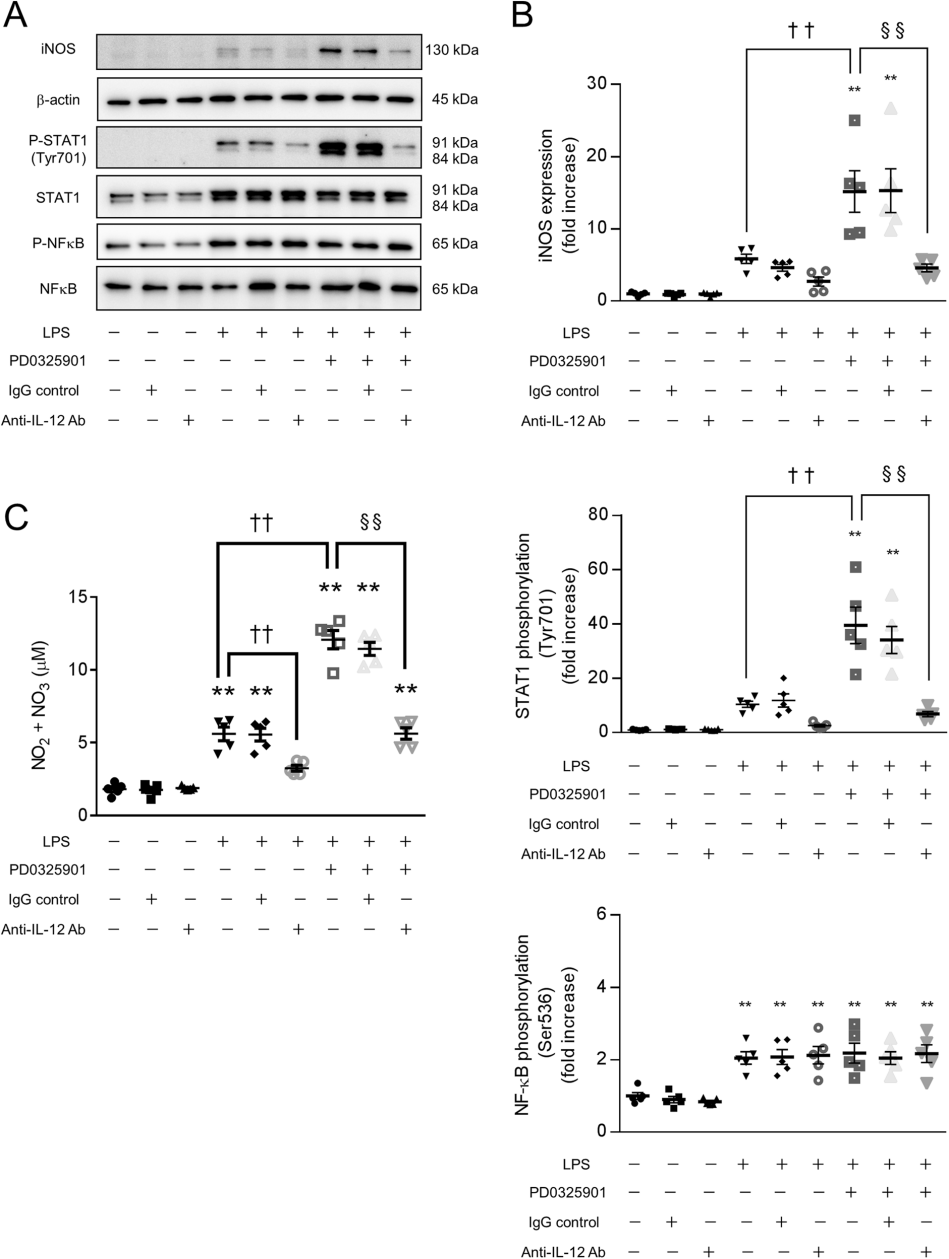
The increases in iNOS expression and NO production in LPS-stimulated macrophages treated with a MEK inhibitor are blocked by an anti-IL-12 neutralizing antibody. BMDMs were treated with the MEK inhibitor PD0325901 for 60 min, followed by stimulation with LPS and the indicated concentrations of anti-IL-12 neutralizing antibodies or isotype control antibodies (anti-trinitrophenol antibody) for 300 min (**A**, **B**) or overnight (**C**). **A**, **B** The cell extracts were analyzed by western blotting. **C** The concentration of NO in the supernatant is shown as the concentration of NO_2_^−^ + NO_3_^−^. **A**–**C** The LPS-induced increases in STAT1 phosphorylation (Tyr701), iNOS expression, and NO production were blocked by the anti-IL-12 neutralizing antibody. *n* = 5 (**p < 0.01 vs. the control group; ^††^*p* < 0.01 vs. the LPS-treated group; ^§§^*p* < 0.01).

To investigate the importance of IL-12 in iNOS expression and NO production, we examined the effects of an anti-IL-12 neutralizing antibody on the increases in iNOS expression and NO production in LPS-stimulated macrophages treated with a MEK inhibitor. Treatment with anti-IL-12 neutralizing antibody (1 μg/mL) did not affect the LPS-induced phosphorylation of NF-κB (Ser536) (Fig. 6A, B). On the other hand, the LPS-induced phosphorylation of STAT1 (Tyr701) and expression of iNOS were suppressed by the anti-IL-12 neutralizing antibody (Fig. 6A, B). Similarly, LPS-induced NO production was suppressed by the anti-IL-12 neutralizing antibody (Fig. 6C). The IgG2 isotype control (anti-trinitrophenol antibody, 1 μg/mL) did not affect LPS-induced NO production (Fig. 6C).

### The MEK inhibitor enhances LPS-induced NO production and morality rate in mice with inflammation

**Fig. 7:**
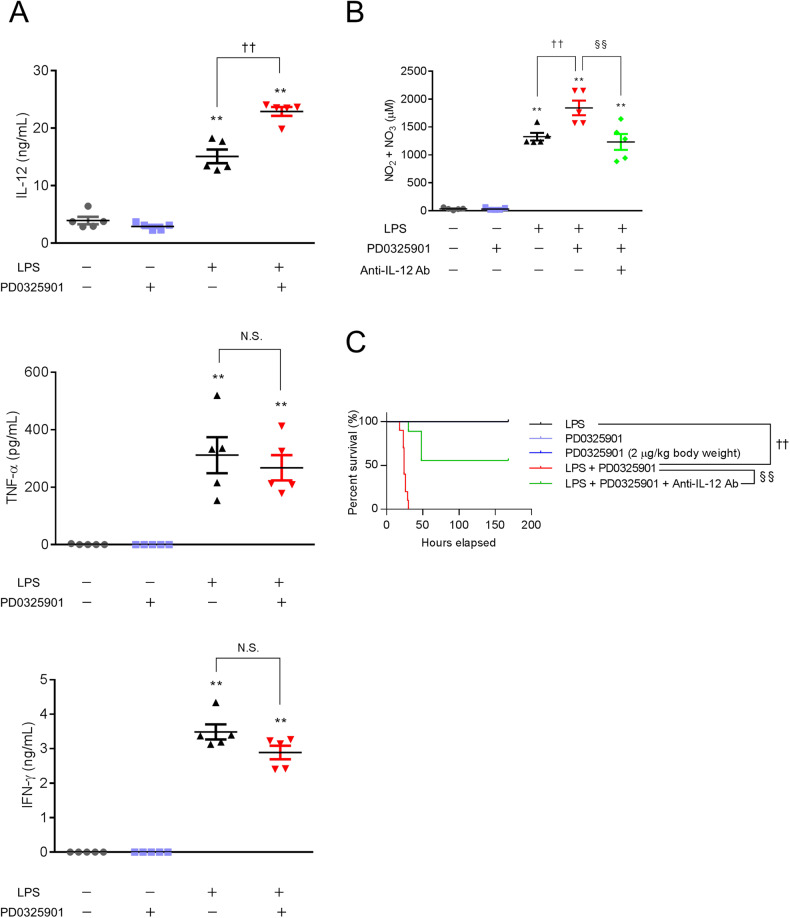
An anti-IL-12 neutralizing antibody prevents NO production and mortality in an LPS-induced inflammation mouse model in the presence of a MEK inhibitor. Mice were intraperitoneally injected with PBS (control), LPS (20 mg/kg body weight), the MEK inhibitor PD0325901 (200 ng/kg body weight or 2 μg/kg body weight), LPS plus PD0325901 (200 ng/kg body weight), or LPS, PD0325901 (200 ng/kg body weight), plus an anti-IL-12 neutralizing antibody (38.8 mg/kg body weight). **A**, **B** Serum was collected 16 h after intraperitoneal injection. **A** Serum concentrations of IL-12, TNF-α, and IFN-γ were determined by ELISA. **B** The concentration of NO in the protein-depleted serum was determined by a method using DAN and is shown as the concentration of NO_2_^−^ + NO_3_^−^. **A**, **B** *n* = 5 (**p < 0.01 vs. the control group; ^††^*p* < 0.01 vs. the LPS-treated group; §§p < 0.01). N.S. means not significant. **C** Survival rates of mice after intraperitoneal injection are shown. *n* = 5 for the PD0325901 (200 ng/kg body weight) and PD0325901 (2 μg/kg body weight) groups, and *n* = 10 for each other group (^††^*p* < 0.01 vs. the LPS-treated group; ^§§^*p* < 0.01).

To verify the in vitro results in vivo, we examined the effects of a MEK inhibitor on LPS-treated mice, which is the most popular inflammation model [29]. In our experiment, injection of 40 mg/kg body weight LPS induced 100% mortality (data not shown); on the other hand, injection of 20 mg/kg body weight LPS or the MEK inhibitor PD0325901 (200 ng/kg body weight or 2 μg/kg body weight) did not result in mortality (Fig. 7C). However, injection of both LPS (20 mg/kg body weight) and the MEK inhibitor PD0325901 (200 ng/kg body weight) induced mortality in 100% of the mice by 48 h (Fig. 7C). Injection of LPS (20 mg/kg body weight) increased NO production in mice, which was enhanced by the MEK inhibitor PD0325901 (200 ng/kg body weight) (Fig. 7B). These results suggest that the MEK inhibitor increases NO production and the mortality rate in an LPS-induced inflammation mouse model.

### An anti-IL-12 neutralizing antibody prevents NO production and mortality in an LPS-induced inflammation mouse model in the presence of a MEK inhibitor

When we measured the levels of L-12, TNF-α, and IFN-γ in mouse serum, we observed increased levels of only IL-12 after stimulation with LPS and a MEK inhibitor (Fig. 7A). Therefore, we examined the effects of an anti-IL-12 neutralizing antibody on NO production and LPS-induced lethality in mice. Injection of LPS (20 mg/kg body weight) increased NO production in mice, which was enhanced by with the MEK inhibitor PD0325901 (200 ng/kg body weight) (Fig. 7B). An anti-IL-12 neutralizing antibody (38.8 mg/kg body weight) prevented NO production in the LPS-induced inflammation mouse model after MEK inhibitor treatment (Fig. 7B). LPS plus PD0325901 induced mortality in 100% of the mice by 48 h (Fig. 7C). An anti-IL-12 neutralizing antibody prevented mortality in the LPS-induced inflammation mouse model after MEK inhibitor treatment (Fig. 7C). These results suggest that MEK inhibitors increase the mortality rate in the LPS-induced inflammation model through IL-12-NO signaling.

## Discussion

In the present study, we showed that MEK inhibitors enhanced iNOS expression and NO production in LPS-stimulated mouse bone marrow-derived macrophages and induced excessive NO production and a high mortality rate in mice with LPS-induced inflammation. An antibody array and ELISA were used to identify cytokines whose expression was enhanced by MEK inhibitors. IL-12 production was markedly enhanced in the culture supernatant of macrophages and in the serum of inflammation model mice during stimulation with LPS and a MEK inhibitor. IL-12 enhanced iNOS expression and NO production in response to LPS. We also showed that TNF-α secretion was induced by stimulation with LPS in macrophages and that TNF-α and IL-12 synergistically induced iNOS expression and NO production. An anti-IL-12 neutralizing antibody suppressed the increases in iNOS expression and NO production in LPS-stimulated macrophages treated with a MEK inhibitor in vitro and prevented LPS-induced lethality by reducing serum NO levels in the mouse model of inflammation after MEK inhibitor treatment. These results suggest that the MEK inhibitor increases the mortality rate in an LPS-induced inflammation model through IL-12-NO signaling.

**Fig. 8:**
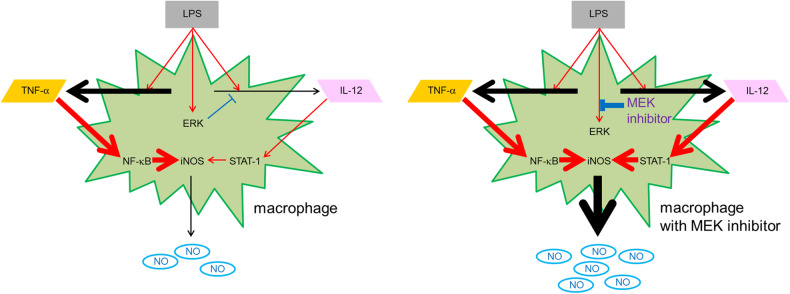
Schematic overview of macrophage treatment with MEK inhibitors. Macrophages are the major producers of IL-12 and TNF-α in response to exogenous or endogenous signals, including LPS. In the present study, we revealed that IL-12 increases iNOS expression and NO production in macrophages in the presence of TNF-α in vitro. We suggest that macrophages treated with MEK inhibitors produce abundant NO through IL-12-STAT1 signaling, which results in an increased mortality rate in an LPS-induced inflammation model.

We observed the secretion of IL-12 and TNF-α but not IFN-γ in LPS-induced mouse bone marrow-derived macrophages. We identified IL-12 as a pivotal cytokine that enhances iNOS expression and NO production in collaboration with TNF-α. Recently, it was reported that IL-12 increases iNOS expression in vivo [30]. However, it has not been revealed whether IL-12 alone increases iNOS expression because there are many cytokines in vivo. IL-12 plus IFN-γ have been reported to increase iNOS expression in vitro [31]. In the present study, the increases in iNOS expression and NO production induced by TNF-α were synergistically strengthened by the addition of IL-12, whereas these effects were not observed in response to IL-12 alone. Unlike IFN-γ, macrophages are the major producers of TNF-α [32]. These findings suggest that IL-12, which is also produced predominantly by macrophages [32], increases iNOS expression and NO production in combination with TNF-α (Fig. 8).

We showed that iNOS expression was enhanced by MEK inhibitors in LPS-stimulated macrophages. Our data are consistent with a prior study [33]; however, opposite effects of MEK inhibitors on iNOS expression have been reported [34–36]. This conflicting effect of MEK inhibitors could be explained by the balance between enhancing iNOS expression by increasing IL-12 concentrations and suppressing iNOS expression by decreasing TNF-α concentrations. In the present study, we showed that IL-12 and TNF-α synergistically induced iNOS expression and NO production in macrophages. We showed that IL-12 production was enhanced by MEK inhibitors in LPS-stimulated macrophages and in an LPS-induced inflammation mouse model, which is consistent with prior studies [34, 37–39]. On the other hand, MEK inhibitors have been reported to suppress the production of TNF-α in LPS-stimulated macrophages [40, 41]. In our study conditions, MEK inhibitors slightly suppressed TNF-α production, but the effect was not significant in vitro or in vivo, and so overall iNOS expression and NO production may have been increased in LPS-stimulated macrophages. We suggest that the balance between IL-12 and TNF-α concentrations might contribute to iNOS expression and NO production in macrophages.

IL-12 and IL-23 share a common p40 (40 kDa) subunit [42]. IL-23 activates many of the same effector cell subtypes [43], and along with IL-12, its expression is increased in several immunological diseases, including psoriasis [44] and Crohn’s disease [45, 46]. The link between psoriasis and cardiovascular diseases (CVs) is well established [47]. Advances in our understanding of the molecular patterns involved in the complex pathogenesis of psoriasis have shown a strong correlation with atherosclerosis. In the past decade, the management of psoriasis patients has been revolutionized by the introduction of biological therapies, such as anti-TNF-α antibody and anti-IL-12/23 antibody (anti-IL-12 antibody) treatment. An anti-TNF-α antibody may reduce CV events in psoriasis patients, whereas the ability of an anti-IL-12 antibody to reduce CV events remains to be clarified. It has been reported that the expression of iNOS can cause the progression of atherosclerosis [48, 49] and impair NO-dependent relaxation [50]. In the present study, we showed that IL-12 enhanced iNOS expression and NO production in macrophages in the presence of TNF-α. Although safety concerns have been raised regarding the possibility of an increased risk of major adverse cardiovascular events (MACEs) with the use of anti-IL-12 antibodies alone [51], cotreatment with anti-TNF-α and anti-IL-12 antibodies may exert beneficial effects on atherosclerotic development by suppressing NO production. Another study based on echocardiographic data confirmed improvements on myocardial function in 18 psoriatic subjects treated with anti-TNF-α and anti-IL-12 antibodies [52].

It has recently been reported that intravenous ustekinumab, which is an anti-IL-12 antibody, induces response and remission in patients with moderately to severely active Crohn’s disease, an inflammatory bowel disease (IBD) that is refractory to TNF-α antagonists [53]. The benefits of ustekinumab in inducing a response were observed as early as 3 weeks. This prompt onset of clinical efficacy, which is paralleled by decreases in C-reactive protein (CRP) levels, suggests that combined targeting of TNF-α and IL-12 is desirable in highly symptomatic patients who might have enhanced iNOS expression and NO production in macrophages. In fact, the importance of iNOS in genetic susceptibility to younger IBD presentation due to higher NO production has been reported [54]. A deleterious role of NO in IBD was proposed after clinical studies reported the presence of high levels of nitrite/nitrate in plasma, urine, and the lumen of the colon [55]. Moreover, a correlation between the overexpression of iNOS, increased concentrations of NO, and the severity of diseases was shown [56]. While it would be ideal to target iNOS itself, human-specific iNOS inhibitors have not been established for clinical use. Thus, directly targeting these cytokines may represent the most efficient therapeutic strategy to pursue, since anti-TNF-α and anti-IL-12 antibodies are already approved for clinical use.

In summary, we showed that TNF-α and IL-12 synergistically induce iNOS expression and NO production in macrophages. We also revealed that the MEK inhibitor increased the mortality rate in mice with LPS-induced inflammation through IL-12-NO signaling.

## Materials and methods

### Experimental animals

All animal care and experimental procedures complied with the Guide for the Care and Use of Laboratory Animals published by the US National Institutes of Health. The experimental protocol was approved by the Animal Care and Use Committee of Juntendo University. The experimental procedures were carried out on male C57BL/6N mice (Sankyo Labo Service Corporation, Tokyo, Japan), ranging from 8 to 12 weeks of age and weighing 20–28 g at the beginning of the study. The mice were maintained under a 12:12 h light–dark cycle with free access to water and food under controlled environmental conditions (21–25 °C and 40–60% relative humidity). The animals were allowed to acclimate for 1 week before the investigation.

### Cell culture

Male C57BL/6N mice at 8–12 weeks of age were euthanized by cervical dislocation, and bone marrow cells were collected from the tibia and femur. The cells were cultured for 6–8 days in RPMI 1640 (Fujifilm Wako Pure Chemical Corporation, Osaka, Japan) containing 10% fetal bovine serum (FBS, Quitech-Bio, TX, USA) and 20 ng/mL M-CSF (Fujifilm Wako Pure Chemical Corporation) at 37 °C in 5% CO_2_/95% air, and primary mouse bone marrow-derived macrophages were generated. Bone marrow-derived macrophages were stimulated with the following reagents unless otherwise noted: LPS (100 ng/mL LPS from *Escherichia coli* O111:B4, Sigma‒Aldrich, MO, USA), IL-12 (10 or 100 ng/mL, PeproTech, NJ, USA), TNF-α (10 ng/mL or 100 ng/mL, R&D Systems, MN, USA), the JAK-STAT inhibitor ruxolitinib (1 μM, Selleck Chemicals, TX, USA), the MEK inhibitor U0126 (1 μM, Fujifilm Wako Pure Chemical Corporation), the MEK inhibitor PD0325901 (10 nM, Fujifilm Wako Pure Chemical Corporation), an anti-IL-12 neutralizing antibody (100 ng/mL or 1 μg/mL, Bio X Cell, NH, USA, clone C17.8, Cat# BE0051, RRID: AB_1107698), and an anti-trinitrophenol antibody (IgG2 isotype control, 1 μg/mL, Bio X Cell, clone 2A3, Cat# BE0089, RRID: AB_1107769).

### Intraperitoneal injection (in vivo)

Male C57BL/6 mice (8 weeks of age) were intraperitoneally administered phosphate-buffered saline (PBS), LPS (20 mg/kg body weight), PD0325901 (200 ng/kg body weight or 2000 ng/kg body weight), LPS plus PD0325901, or LPS, PD0325901, plus the anti-IL-12 neutralizing antibody (38.8 mg/kg body weight).

### Immunoblotting

After being treated, the cells were washed two times with PBS and lysed in 100 μl of RIPA buffer with a protease and phosphatase inhibitor cocktail and EDTA (Thermo Fisher Scientific, MA, USA). The protein concentration of the cell lysate was determined with a BCA Protein Assay Kit (Thermo Fisher Scientific). The cell extract was diluted in 4× sample buffer (Bio-Rad Laboratories, CA, USA) and then boiled for 5 min at 95 °C. Equal amounts of proteins were loaded onto an SDS-10% polyacrylamide gel and resolved by one-dimensional SDS‒PAGE at a constant current of 10 mA/gel (Rapidas Mini Slab Gel Electrophoresis Apparatus, ATTO Corporation, Tokyo, Japan). After the gel was equilibrated in transfer buffer (25 mM Tris, 192 mM glycine, and 15% methanol), the proteins were electrophoretically transferred onto a polyvinylidene difluoride (PVDF) membrane (Merck Millipore Corporation, Darmstadt, Germany) at 100 V for 40 min on ice (Mini Trans-Blot Electrophoretic Transfer Cell, Bio-Rad Laboratories). The membrane was blocked with PVDF blocking reagent (Toyobo Co., Ltd., Osaka, Japan) and incubated with appropriate dilutions of the primary antibodies in Can Get Signal Solution 1 (Toyobo Co., Ltd.) at 4 °C overnight. The following primary antibodies were purchased from Cell Signaling Technology (MA, USA): iNOS (D6B6S) rabbit monoclonal antibody (1:2000, Cat# 13120, RRID: AB_2687529), β-actin (D6A8) rabbit monoclonal antibody (1:10,000, Cat# 8457, RRID: AB_10950489), ERK1/2 (137F5) rabbit monoclonal antibody (1:2000, Cat# 4695, RRID: AB_390779), phospho-ERK1/2 (Thr202/Tyr204) (D13.14.4E) rabbit monoclonal antibody (1:2000, Cat# 4370, RRID: AB_2315112), STAT1 (D1K9Y) rabbit monoclonal antibody (1:2000, Cat# 14994, RRID: AB_2737027), phospho-STAT1 (Tyr701) (D4A7) rabbit monoclonal antibody (1:2000, Cat# 7649, RRID: AB_10950970), phospho-STAT1 (Ser727) rabbit monoclonal antibody (1:2000, Cat# 9177, RRID: AB_2197983), NF-κB p65 (D14E12) rabbit monoclonal antibody (1:2000, Cat# 8242, RRID: AB_10859369), and phospho-NF-κB p65 (Ser536) (93H1) rabbit monoclonal antibody (1:2000, Cat# 3033, RRID: AB_331284). After the membrane was washed with Tris-buffered saline and 0.1% Tween-20 (TBS-T), it was incubated for 1 h at room temperature with an anti-rabbit IgG horseradish peroxidase-conjugated secondary antibody (Cell Signaling Technology, 1:1000, Cat# 7074, RRID: AB_2099233) in Secondary Antibody Solution 2 (Toyobo Co., Ltd.). The blots were detected using ECL Prime reagent (Cytiva, MA, USA), and signals were obtained with a luminescent image analyzer (ImageQuant LAS 4000, GE Healthcare, NJ, USA). Analyses were performed using ImageJ software (version 1.47 v, US National Institutes of Health, MD, USA). The expression of iNOS was normalized to β-actin expression, and the phosphorylation of ERK, STAT1, and NF-κB p65 was normalized to the basal expression of each protein.

### NO measurement

After overnight treatment with reagents in phenol red-free DMEM (Fujifilm Wako Pure Chemical Corporation), the cell culture medium was collected and centrifuged at 1000 × *g* for 15 min, and the supernatant was used as a sample solution. At 16 h after the intraperitoneal injection of reagents, blood was taken from the mouse hearts. Then, the blood was centrifuged at 10,000 × *g* for 2 min to separate the serum using a BD Microtainer Tube (Becton, Dickinson and Company, NJ, USA). The serum was centrifuged with an Amicon Ultra4 Centrifugal Filter Unit with an Ultracel-10 membrane (Merck, Darmstadt, Germany) at 14,000 × *g* for 15 min to remove proteins, and the protein-free serum was used as a sample solution. The combined concentrations of NO_2_^−^ and NO_3_^−^, which are the degradation products of NO, were measured using a NO_2_/NO_3_ Assay Kit (Dojindo Laboratories, Kumamoto, Japan) and a plate reader system (FlexStation 3, Molecular Devices, CA, USA). Total NO_2_^−^/NO_3_^−^ production indicates NO production.

### Antibody array

After treatment with reagents, the cell culture medium was collected and centrifuged at 1000 × *g* for 15 min, and the supernatant was used as a sample solution. To detect 111 mouse cytokines (Adiponectin/Acrp30, Amphiregulin, Angiopoietin-1, Angiopoietin-2, Angiopoietin-like 3, BAFF/BLyS/TNFSF13B, C1q R1/CD93, CCL2/JE/MCP-1, CCL3/CCL4/MIP-1α/β, CCL5/RANTES, CCL6/C10, CCL11/Eotaxin, CCL12/MCP-5, CCL17/TARC, CCL19/MIP-3β, CCL20/MIP-3α, CCL21/6Ckine, CCL22/MDC, CD14, CD40/TNFRSF5, CD160, Chemerin, Chitinase 3-like 1, Coagulation Factor III/Tissue Factor,Complement Component C5/C5a, Complement Factor D, C-Reactive Protein/CRP, CX3CL1/Fractalkine, CXCL1/KC, CXCL2/MIP-2, CXCL9/MIG, CXCL10/IP-10, CXCL11/I-TAC, CXCL13/BLC/BCA-1, CXCL16, Cystatin C, Dkk-1, DPPIV/CD26, EGF, Endoglin/CD105, Endostatin, Fetuin A/AHSG, FGF acidic, FGF-21, Flt-3 Ligand, Gas6, G-CSF, GDF-15, GM-CSF, HGF, ICAM-1/CD54,IFN-γ, IGFBP-1, IGFBP-2, IGFBP-3, IGFBP-5, IGFBP-6, IL-1α/IL-1F1, IL-1β/IL-1F2, IL-1λ/IL-1F3, IL-2, IL-3, IL-4,IL-5, IL-6, IL-7, IL-10, IL-11, IL-12p40, IL-13, IL-15, IL-17A, IL-22, IL-23, IL-27p28, IL-28A/B, IL-33, LDLR, Leptin, LIF, Lipocalin-2/NGAL, LIX, M-CSF, MMP-2, MMP-3, MMP-9, Myeloperoxidase, Osteopontin (OPN), Osteoprotegerin/TNFRSF11B, PD-ECGF/Thymidine phosphorylase, PDGF-BB, Pentraxin 2/SAP, Pentraxin 3/TSG-14, Periostin/OSF-2, Pref-1/DLK-1/FA1, Proliferin, Proprotein Convertase 9/PCSK9, RAGE, RBP4, Reg3G, Resistin, E-Selectin/CD62E, P-Selectin/CD62P, Serpin E1/PAI-1, Serpin F1/PEDF, Thrombopoietin, TIM-1/KIM-1/HAVCR, TNF-α, VCAM-1/CD106, VEGF, and WISP-1/CCN4), the sample solution was analyzed using a Proteome Profiler Mouse XL Cytokine Array Kit (R&D Systems) according to the manufacturer’s instructions. The signals were detected with a luminescent image analyzer (ImageQuant LAS 4000, GE Healthcare). Analyses were performed using ImageJ software.

### ELISA

After treatment with reagents, the cell culture medium was collected centrifuged at 1000 × *g* for 15 min, and the supernatant was used as a sample solution. At 16 h after the intraperitoneal injection of reagents, blood was collected from the mouse heart. Then, the blood was centrifuged at 10,000 × *g* for 2 min to separate the serum using a BD Microtainer Tube, and the serum was used as a sample solution. The concentrations of IL-12, TNF-α, and IFN-γ were measured using an ELISA kit (Fujifilm Wako Shibayagi Corporation, Gunma, Japan) and a plate reader system (Benchmark Plus, Bio-Rad Laboratories).

### Survival rate of mice after LPS administration

Mouse survival was monitored over a period of 168 h after the intraperitoneal injection of reagents.

### Data and statistical analysis

Cells and mice were randomly assigned to each treatment group and experiment. GraphPad Prism 6 software (GraphPad Software, CA, USA) was used for data analysis. The data are expressed as the mean ± standard error of the mean (SEM). Statistical significance was determined by one-way ANOVA for three or more groups. Survival curves were compared using the log-rank (Mantel‒Cox) test. A probability level of *p* < 0.05 was considered statistically significant.

### Supplementary information

Original Western Blots

## Data Availability

The experimental data sets generated and/or analyzed during the current study are available from the corresponding author upon reasonable request. No applicable resources were generated during the current study.
