# Supplementary material for: MEK inhibitors increase the mortality rate in mice with LPS-induced inflammation through IL-12-NO signaling
Source: Cell Death Discov. 2023 Oct 13;9:374. doi: 10.1038/s41420-023-01674-w (PMC10575927; doi:10.1038/s41420-023-01674-w)
Supplement: Supplementary file 1 — Original Western Blots [file 41420_2023_1674_MOESM1_ESM.pdf]

Figure 1A

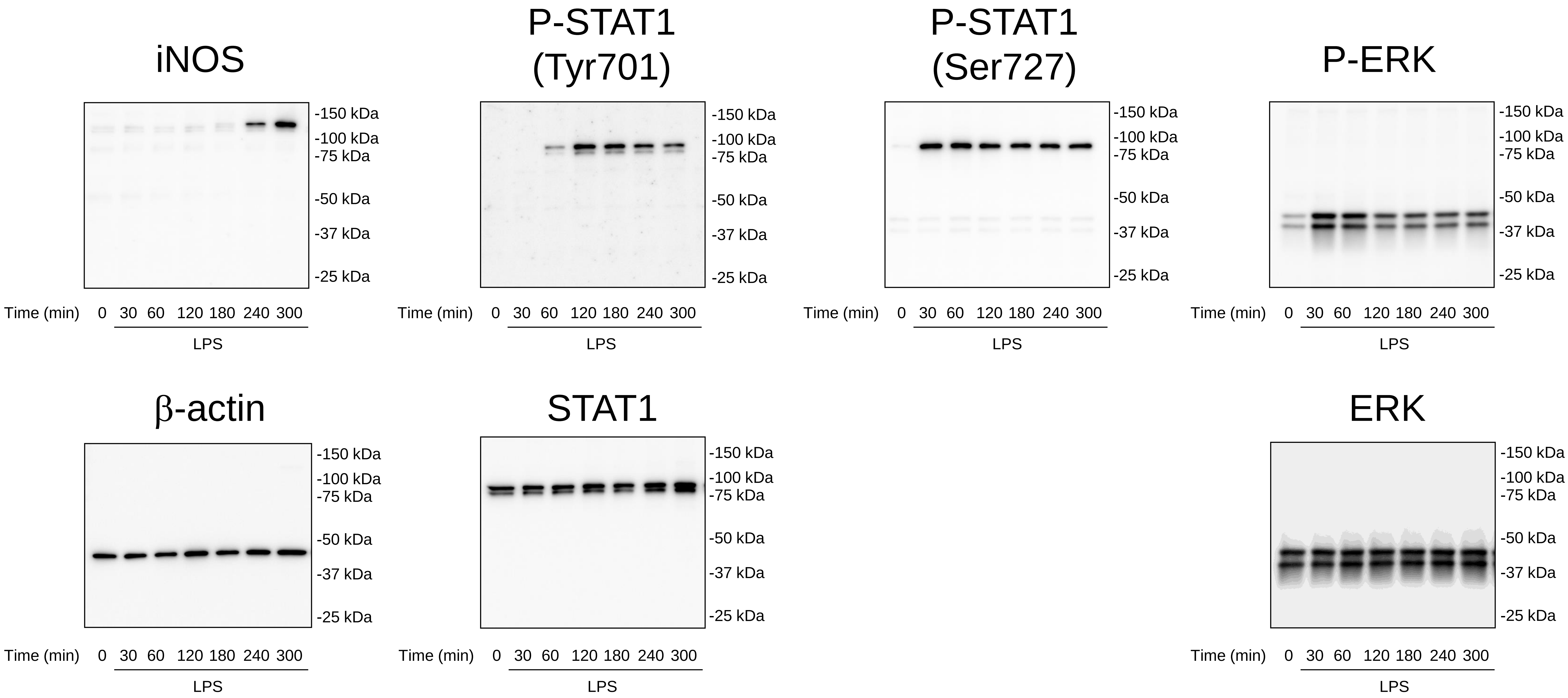

iNOS
P-STAT1 (Tyr701)
P-STAT1 (Ser727)
P-ERK
β-actin
STAT1
ERK
-150 kDa
-100 kDa
-75 kDa
-50 kDa
-37 kDa
-25 kDa
Time (min) 0 30 60 120 180 240 300
LPS

Figure 1C

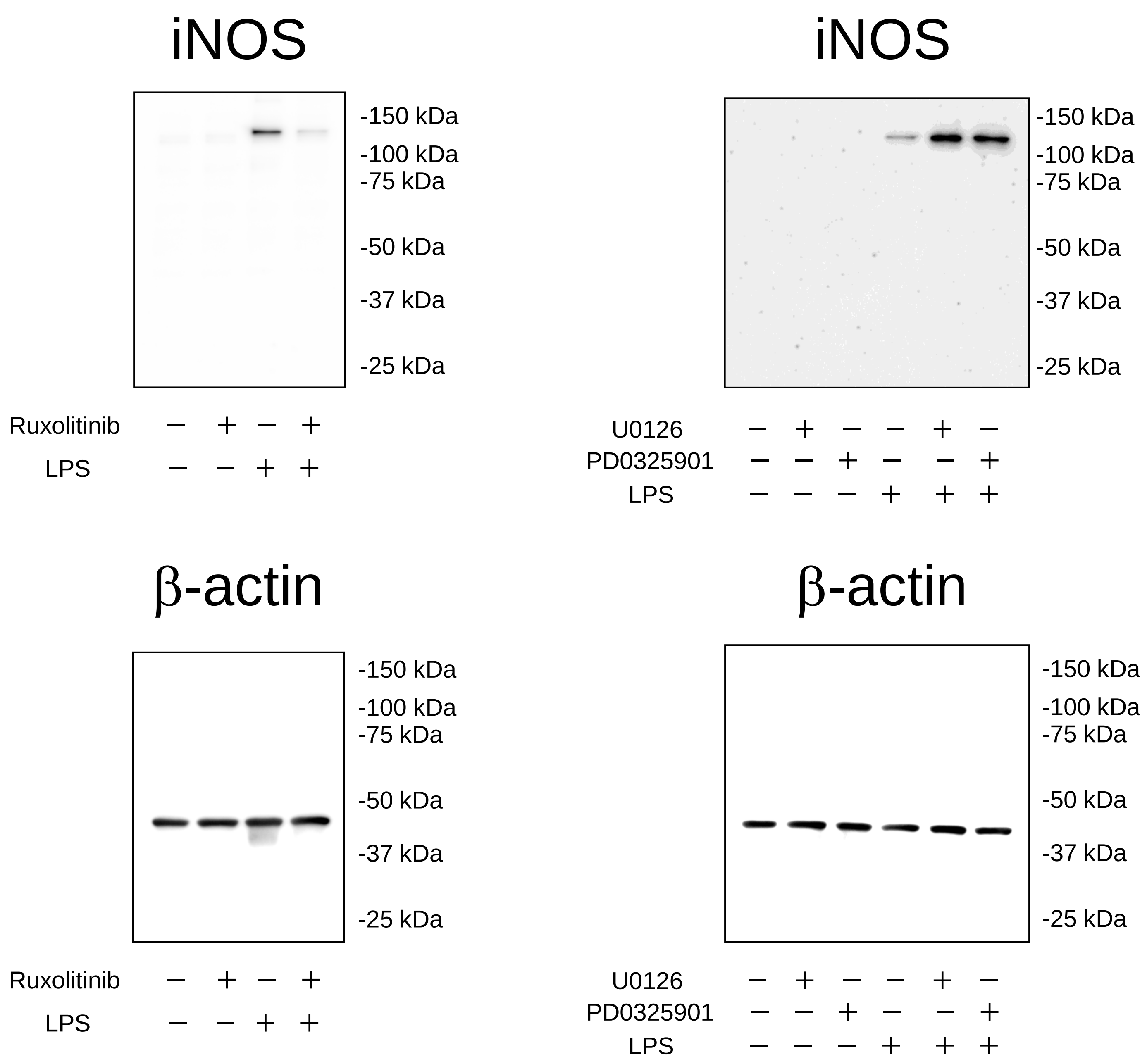
iNOS
-150 kDa
-100 kDa
-75 kDa
-50 kDa
-37 kDa
-25 kDa
Ruxolitinib − + − +
LPS − − + +
iNOS
-150 kDa
-100 kDa
-75 kDa
-50 kDa
-37 kDa
-25 kDa
U0126 − + − − + −
PD0325901 − − + − − +
LPS − − − + + +
β-actin
-150 kDa
-100 kDa
-75 kDa
-50 kDa
-37 kDa
-25 kDa
Ruxolitinib − + − +
LPS − − + +
β-actin
-150 kDa
-100 kDa
-75 kDa
-50 kDa
-37 kDa
-25 kDa
U0126 − + − − + −
PD0325901 − − + − − +
LPS − − − + + +

Figure 2A-1

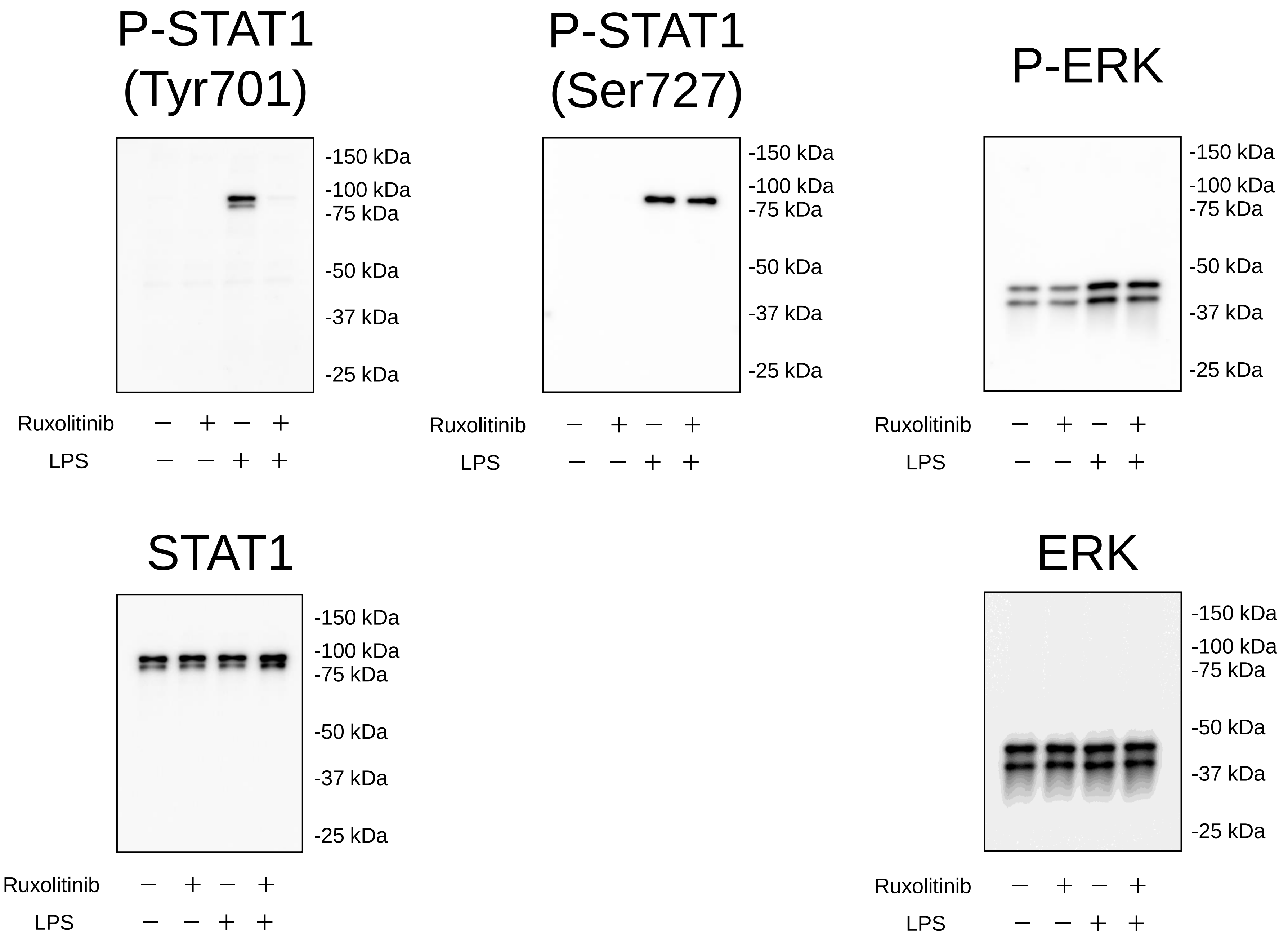
P-STAT1 (Tyr701)
-150 kDa
-100 kDa
-75 kDa
-50 kDa
-37 kDa
-25 kDa
Ruxolitinib − + − +
LPS − − + +
P-STAT1 (Ser727)
-150 kDa
-100 kDa
-75 kDa
-50 kDa
-37 kDa
-25 kDa
Ruxolitinib − + − +
LPS − − + +
P-ERK
-150 kDa
-100 kDa
-75 kDa
-50 kDa
-37 kDa
-25 kDa
Ruxolitinib − + − +
LPS − − + +
STAT1
-150 kDa
-100 kDa
-75 kDa
-50 kDa
-37 kDa
-25 kDa
Ruxolitinib − + − +
LPS − − + +
ERK
-150 kDa
-100 kDa
-75 kDa
-50 kDa
-37 kDa
-25 kDa
Ruxolitinib − + − +
LPS − − + +

Figure 2A-2

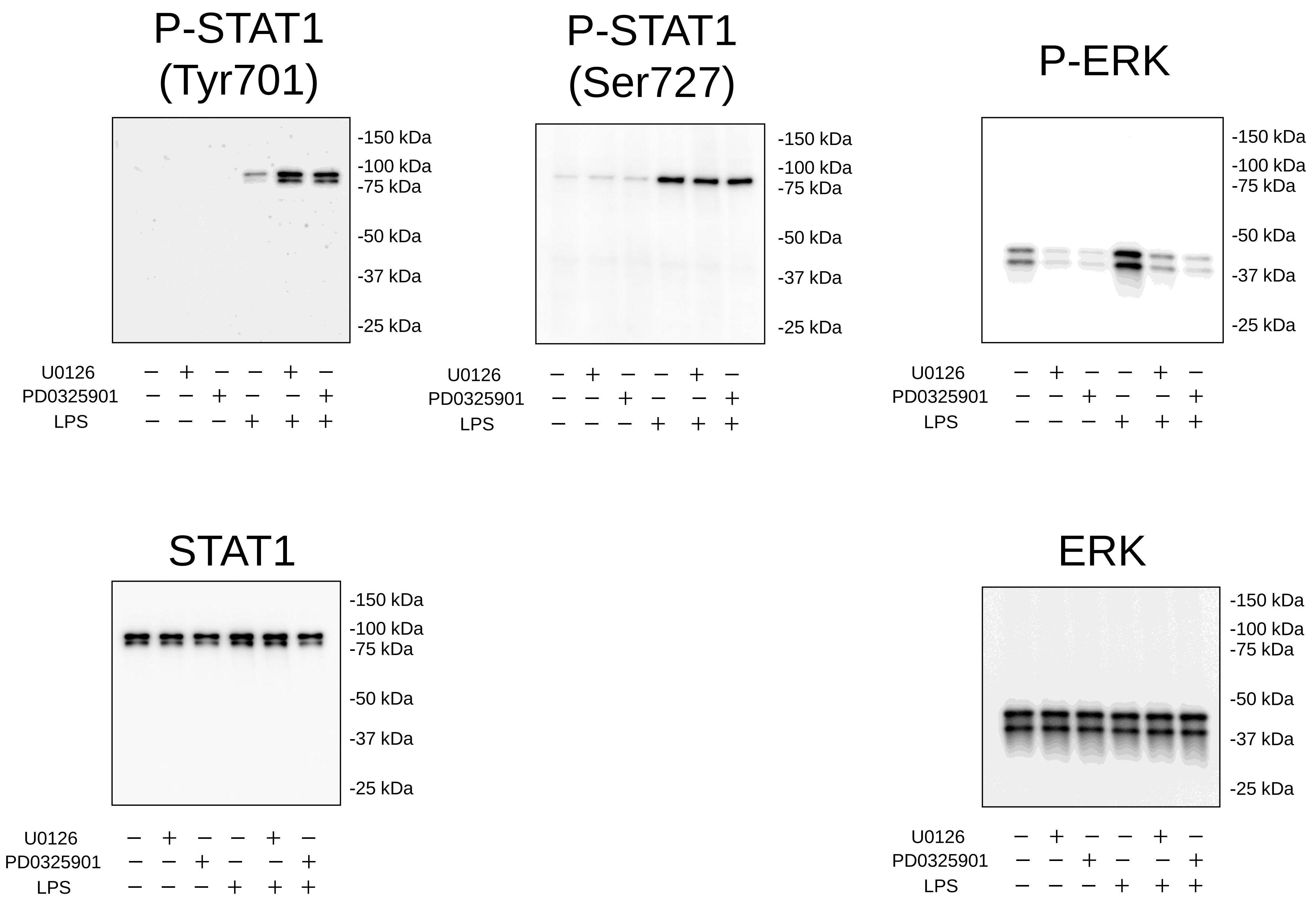
P-STAT1 (Tyr701)
P-STAT1 (Ser727)
P-ERK
STAT1
ERK
-150 kDa
-100 kDa
-75 kDa
-50 kDa
-37 kDa
-25 kDa
U0126 − + − − + −
PD0325901 − − + − − +
LPS − − − + + +

Figure 2C

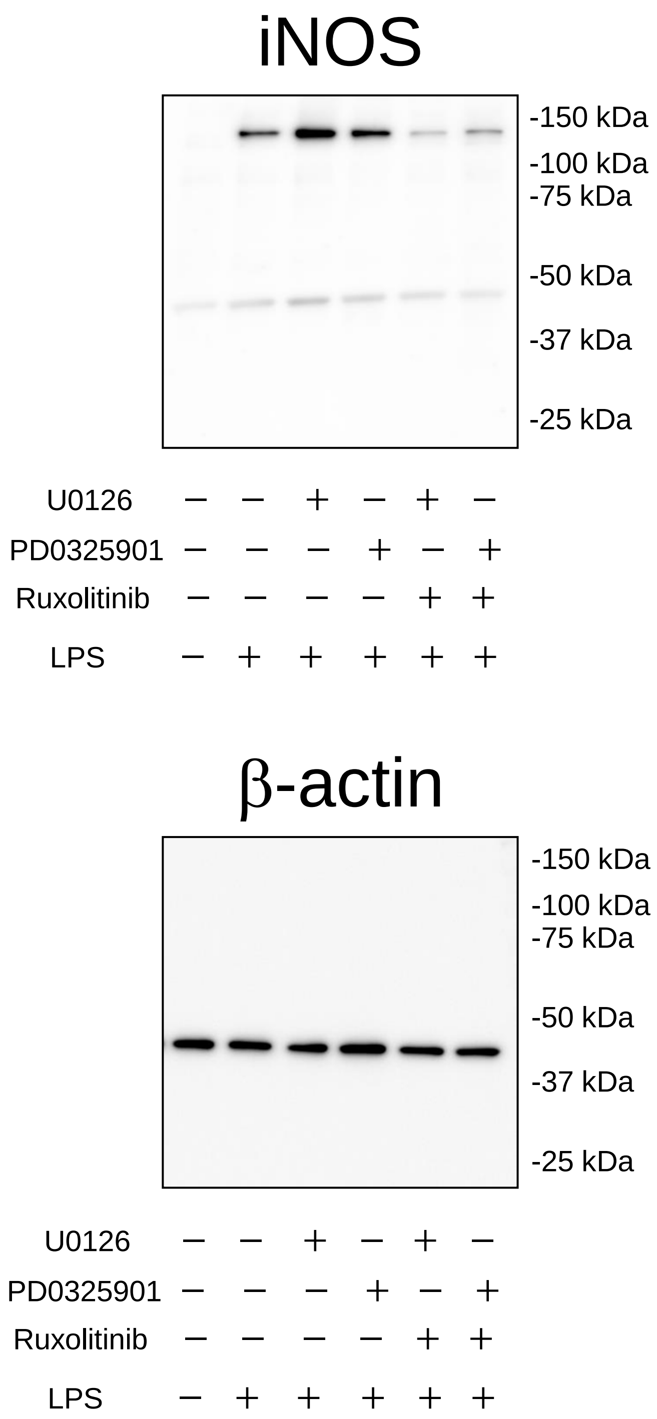
iNOS
-150 kDa
-100 kDa
-75 kDa
-50 kDa
-37 kDa
-25 kDa
U0126 – – + – + –
PD0325901 – – – + – +
Ruxolitinib – – – – + +
LPS – + + + + +
β-actin
-150 kDa
-100 kDa
-75 kDa
-50 kDa
-37 kDa
-25 kDa
U0126 – – + – + –
PD0325901 – – – + – +
Ruxolitinib – – – – + +
LPS – + + + + +

Figure 4

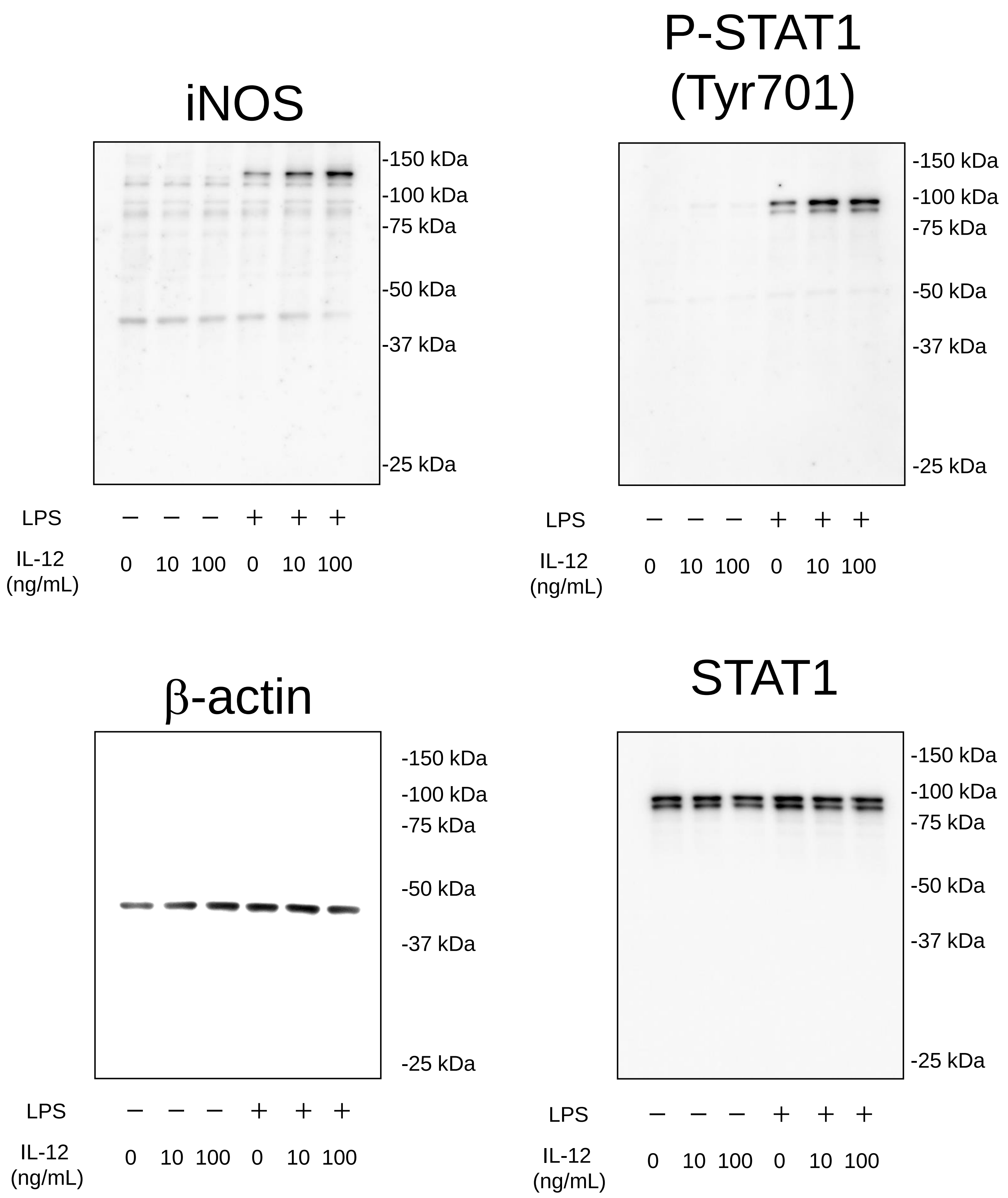

iNOS
-150 kDa
-100 kDa
-75 kDa
-50 kDa
-37 kDa
-25 kDa
LPS – – – + + +
IL-12 (ng/mL) 0 10 100 0 10 100
P-STAT1 (Tyr701)
-150 kDa
-100 kDa
-75 kDa
-50 kDa
-37 kDa
-25 kDa
LPS – – – + + +
IL-12 (ng/mL) 0 10 100 0 10 100
β-actin
-150 kDa
-100 kDa
-75 kDa
-50 kDa
-37 kDa
-25 kDa
LPS – – – + + +
IL-12 (ng/mL) 0 10 100 0 10 100
STAT1
-150 kDa
-100 kDa
-75 kDa
-50 kDa
-37 kDa
-25 kDa
LPS – – – + + +
IL-12 (ng/mL) 0 10 100 0 10 100

Figure 5

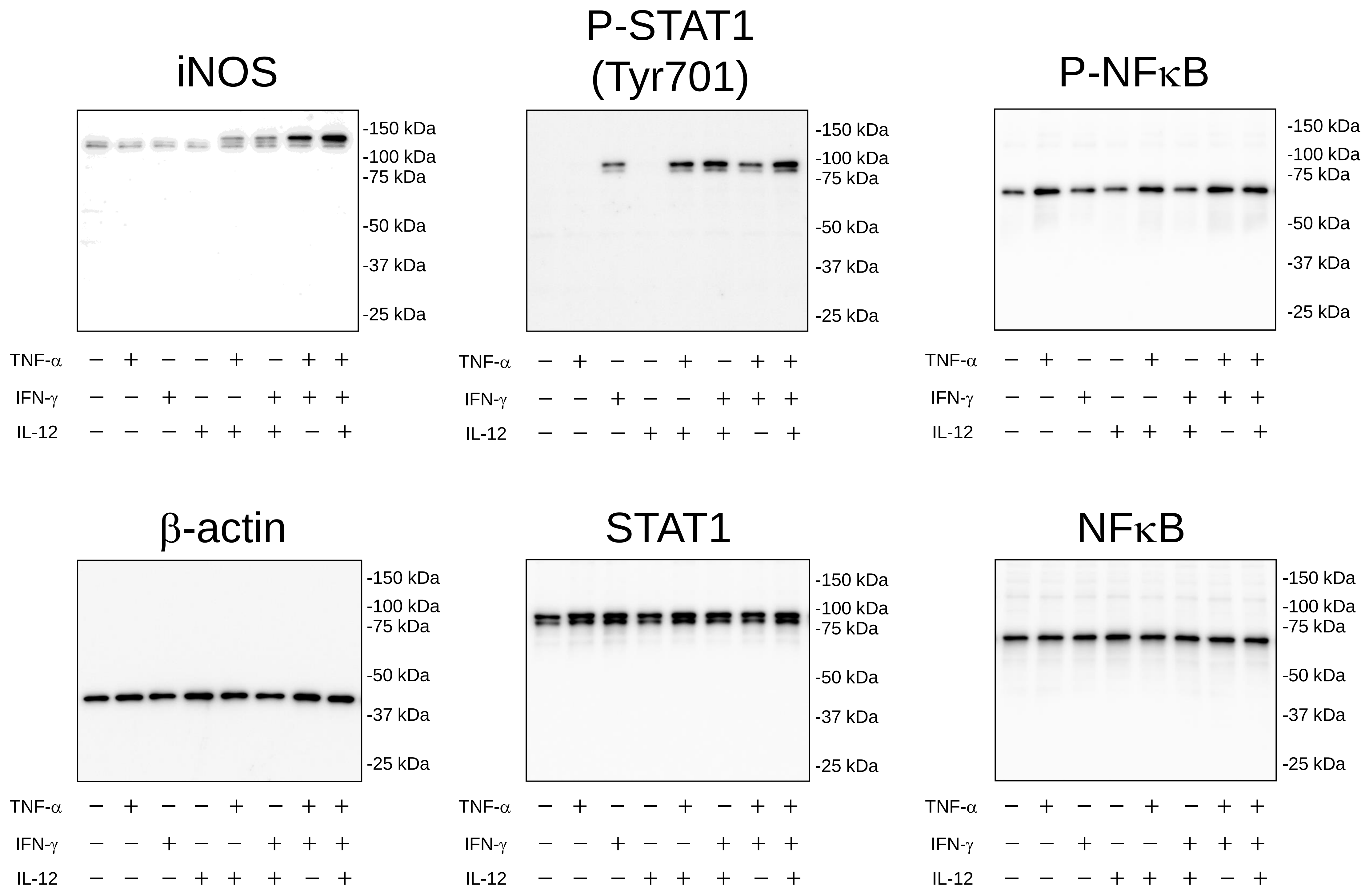
iNOS
P-STAT1 (Tyr701)
P-NFκB
β-actin
STAT1
NFκB
-150 kDa
-100 kDa
-75 kDa
-50 kDa
-37 kDa
-25 kDa
TNF-α − + − − + − + +
IFN-γ − − + − − + + +
IL-12 − − − + + + − +

Figure 6

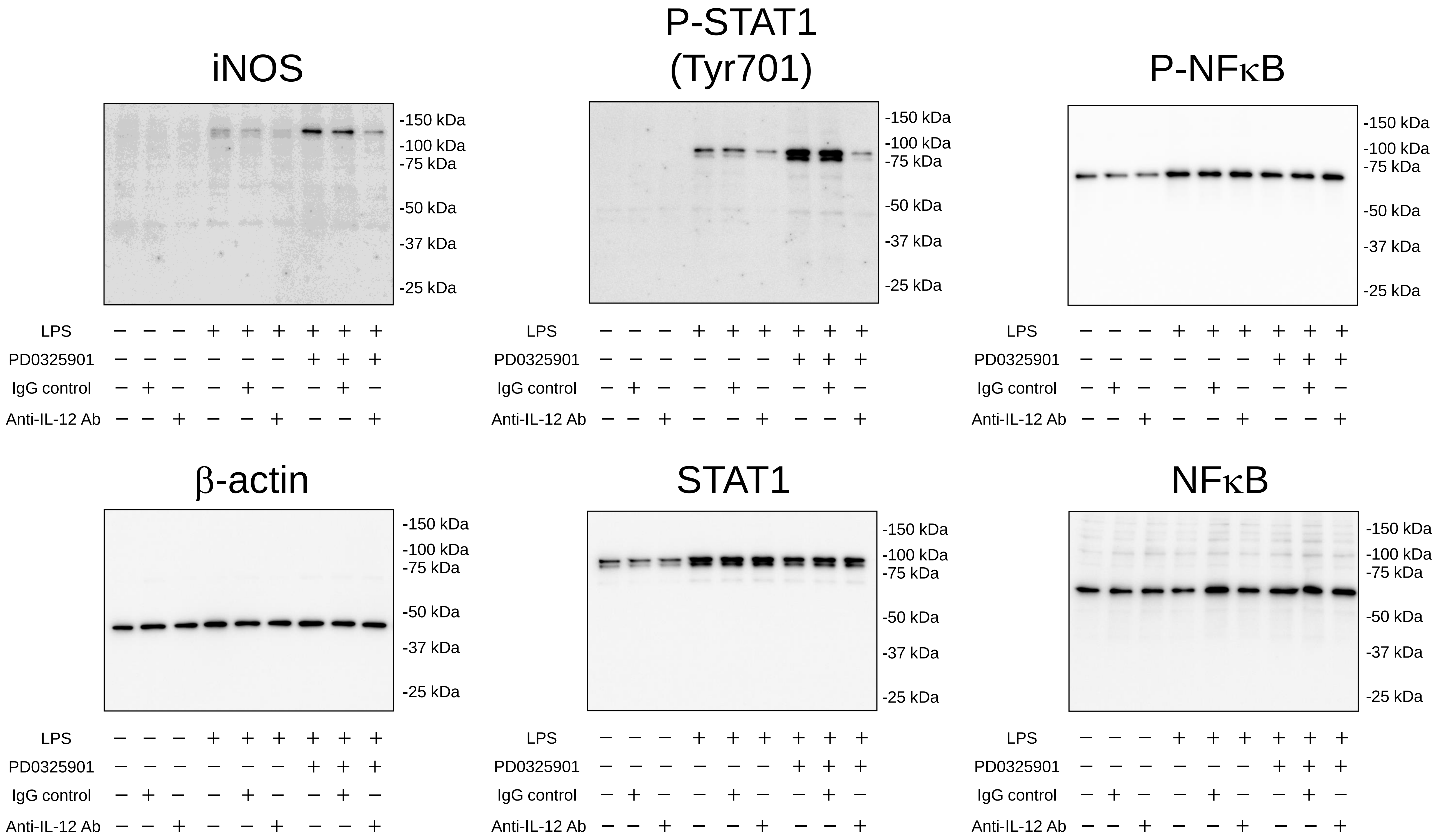
iNOS
P-STAT1 (Tyr701)
P-NFκB
β-actin
STAT1
NFκB
-150 kDa
-100 kDa
-75 kDa
-50 kDa
-37 kDa
-25 kDa
LPS − − − + + + + + +
PD0325901 − − − − − − + + +
IgG control − + − − + − − + −
Anti-IL-12 Ab − − + − − + − − +
